# Tetramethylpyrazine Protects against Hydrogen Peroxide-Provoked Endothelial Dysfunction in Isolated Rat Aortic Rings: Implications for Antioxidant Therapy of Vascular Diseases

**DOI:** 10.1155/2014/627181

**Published:** 2014-09-02

**Authors:** Xiaojia Ni, Siu Ling Wong, Chi Ming Wong, Chi Wai Lau, Xiaogeng Shi, Yefeng Cai, Yu Huang

**Affiliations:** ^1^Guangdong Provincial Hospital of Chinese Medicine, No. 111 Dade Road, Guangzhou 510120, China; ^2^Second College of Clinical Medicine, Guangzhou University of Chinese Medicine, No. 12 Jichang Road, Guangzhou 510405, China; ^3^School of Biomedical Sciences, Chinese University of Hong Kong, Shatin, NT, Hong Kong

## Abstract

*Background and Objectives*. Oxidative stress can initiate endothelial dysfunction and atherosclerosis. This study evaluated whether tetramethylpyrazine (TMP), the predominant active ingredient in Rhizoma Ligustici Wallichii (chuanxiong), prevents endothelial dysfunction in a rat model of oxidative stress. *Methods*. Isolated rat aortic rings were pretreated with various drugs before the induction of endothelial dysfunction by hydrogen peroxide (H_2_O_2_). Changes in isometric tension were then measured in acetylcholine- (ACh-) relaxed rings. Endothelial nitric oxide synthase (eNOS) expression was evaluated in the rings by Western blotting, and superoxide anion (O_2_^∙−^) content was assessed in primary rat aortic endothelial cells by dihydroethidium- (DHE-) mediated fluorescence microscopy. *Results*. ACh-induced endothelium-dependent relaxation (EDR) was disrupted by H_2_O_2_ in endothelium-intact aortic rings. H_2_O_2_-impaired relaxation was ameliorated by acute pretreatment with low concentrations of TMP, as well as by pretreatment with catalase and the NADPH oxidase inhibitors, apocynin and diphenyleneiodonium (DPI). TMP, apocynin, and DPI also reduced O_2_^∙−^ accumulation in endothelial cells,but TMP failed to alter eNOS expression in aortic rings incubated with H_2_O_2_. *Conclusions*. TMP safeguards against oxidative stress-induced endothelial dysfunction, suggesting that the agent might find therapeutic utility in the management of vascular diseases. However, TMP's role in inhibiting NADPH oxidase and its vascular-protective mechanism of action requires further investigation.

## 1. Introduction

Excessive production of reactive oxygen species (ROS) by vascular endothelial cells contributes to endothelial dysfunction [1] which then initiates atherosclerosis [2]. Atherosclerosis is a progressive process that gradually leads to artery blockage. The clinical progression of atherosclerosis culminates in a number of devastating vascular events, including myocardial infarction and stroke [3]. Accordingly, patients with an ischemic complication of atherosclerosis experience a particularly vulnerable disease phase [4]. Currently, prevention and management of cardio-cerebrovascular diseases encompass the control of risk factors, as well as antithrombotic interventions [5, 6]. Additional efforts are directed toward interrupting ongoing oxidative processes in the vessel wall so as to prevent ROS-provoked endothelial impairment [7]. In this regard, antioxidants, which directly remove ROS threats, are emerging as one of the more promising treatments for vascular diseases in the recent years [8].

Rhizoma Ligustici Wallichii (Chinese name, chuanxiong), or lovage tuber, is commonly used in traditional Chinese medicine to promote blood circulation. Tetramethylpyrazine (TMP) is the predominant active component of Rhizoma Ligustici Wallichii [9], and the compound is noted for its potent vasodilating properties [10]. Like the parental herb, TMP (also called ligustrazine) is widely used to treat vascular diseases in China, with few adverse reactions in humans [11]. TMP has a long history of use, and the latest studies report its antioxidant proficiency in animal models of ischemic reperfusion [12], atherosclerosis [13], and cerebral vasospasm [14]. However, the underlying mechanism of TMP to combat detrimental ROS production and to rescue endothelial dysfunction in artery walls remains unclear.

Hydrogen peroxide (H_2_O_2_) acts as a relatively benign cell signaling molecule at low concentrations [15], but exposure of blood vessels to excessive amounts of H_2_O_2_ results in severe endothelial impairment [16]. In addition, H_2_O_2_ released during the progression of atherosclerosis is likely to worsen vascular damage [17]. In light of the protective effect of TMP in clinical and animal studies, we hypothesized that the administration of this compound to H_2_O_2_-compromised blood vessels could prevent endothelial dysfunction. The actions of acute TMP pretreatment on endothelium-dependent relaxation (EDR) and the protein expression levels of endothelial nitric oxide synthase (eNOS) were, therefore, evaluated in isolated rat aortic rings under various experimental conditions. We also explored the preventative effect of TMP against H_2_O_2_-provoked generation of the superoxide anion (O_2_^∙−^), a particularly harmful ROS, in primary cultures of endothelial cells established from the rat aorta.

## 2. Materials and Methods

### 2.1. Drugs and Reagents

TMP was purchased from the National Institutes for Food and Drug Control (Beijing, China). Phenylephrine (Phe), acetylcholine (ACh), apocynin, diphenyleneiodonium (DPI), vitamin E, catalase, Tiron (4,5-dihydroxy-1,3-benzene-disulfonic acid), and diethyldithiocarbamic acid (DETCA) were purchased from Sigma-Aldrich (St. Louis, MO, USA). Dihydroethidium (DHE) was purchased from Molecular Probes (Eugene, OR, USA). Losartan was purchased from Cayman Chemical Co. (Ann Arbor, MI, USA). Apocynin and losartan were dissolved in dimethyl sulfoxide (DMSO), and the other compounds were dissolved in distilled water. The DMSO vehicle did not affect ACh-induced aortic ring relaxation at the concentration employed in this study (0.1% v/v).

### 2.2. Blood Vessel Preparation

All animal experiments were approved by the Animal Experimentation Ethics Committee of the Chinese University of Hong Kong (Approval no. 4362/04 M). Male Sprague Dawley rats aged 6-7 months (260–280 g) were supplied by the Animal Service Center for the Chinese University of Hong Kong and euthanized by carbon dioxide inhalation. Aortas were isolated and dissected as previously described [18].

Briefly, the thoracic segment of each aorta was excised, removed of the surrounding adipose tissue, and cut into rings approximately 3 mm long in ice-cold oxygenated Krebs solution that consisted of 119 mM NaCl, 4.7 mM KCl, 2.5 mM CaCl_2_, 1 mM MgCl_2_, 25 mM NaHCO_3_, 1.2 mM KH_2_PO_4_, and 11 mM D-glucose. The rings were then suspended between stainless stain wires in 10-mL organ baths containing Krebs solution bubbling with 95% O_2_ plus 5% CO_2_ at 37°C. A basal tension of 20 mN was applied to all rings. The rings were exposed to 0.3 *μ*M Phe to test smooth muscle contractility and then were relaxed by the addition of 3 *μ*M ACh to assess the integrity of the endothelium. In some cases, the endothelial layer was mechanically disrupted by gently rubbing the lumen against the tips of a pair of forceps. Removal of the endothelium was confirmed by the lack of relaxation in the presence of 3 *μ*M ACh. The rings were rinsed several times in prewarmed Krebs solution until baseline tension returned and then were used in isometric tension measurement experiments, as described below.

### 2.3. Isometric Tension Measurement

Contraction was once again induced in the aortic rings by exposure to 0.3 *μ*M Phe. A gradient of ACh (3 nM–10 *μ*M) was cumulatively added to induce relaxation of the rings when the contraction response reached a plateau, so as to obtain a concentration-response curve for ACh-mediated relaxation. The rings were again rinsed in prewarmed Krebs solution until baseline tension returned, followed by pretreatment with various compounds, as described below, and exposure to H_2_O_2_ (200 *μ*M, 60 min). Thereafter, a second Ach concentration-response curve was obtained to examine any changes in the EDR imposed by H_2_O_2_ or the drugs.

The first series of experiments was designed to investigate whether TMP pretreatment could rescue endothelial dysfunction provoked by H_2_O_2_. The aortic rings were incubated with various concentrations (100 *μ*M, 1 *μ*M, or 10 nM) of TMP for 60 min and subsequently treated with H_2_O_2_ (200 *μ*M) for 60 min. For comparison, the effects of losartan (1 *μ*M, 30 min), a newly identified antioxidant agent [19], and vitamin E (100 *μ*M, 30 min), a classical antioxidant [20], were also tested.

The next series of experiments was designed to explore the underlying mechanism of H_2_O_2_-induced EDR impairment. The rings were separately pretreated for 30 min with catalase (20000 U/mL), an enzyme that catalyzes the decomposition of H_2_O_2_ to water and molecular oxygen [21], and two inhibitors of NADPH oxidase, apocynin (10 *μ*M) and DPI (10 nM) [22, 23]. After catalase, apocynin, or DPI pretreatment, the rings were exposed to H_2_O_2_ (200 *μ*M) for 60 min.

### 2.4. Western Blotting

Western blotting analysis was performed as previously described [24]. Briefly, aortic rings were snap frozen in liquid nitrogen and homogenized in ice-cold RIPA lysis buffer. The lysates were centrifuged, and the supernatants were collected. Protein concentrations in each sample were determined by the Lowry method. Equal amounts of protein (50 *μ*g per sample) were resolved in 10% sodium dodecyl sulfate- (SDS-) polyacrylamide gels and then transferred to nitrocellulose immobilon-P polyvinylidenedifluoride membranes. The membranes were blocked for 1 h at room temperature with 1% bovine serum albumin and incubated overnight at 4°C with a primary antibody against eNOS (1 : 3000) (BD Transduction Laboratories, Lexington, KY, USA) and a primary antibody against the loading control, glyceraldehyde 3-phosphate dehydrogenase (GAPDH, 1 : 3000) (Ambion, Austin, TX, USA). The membranes were then incubated with the corresponding horseradish peroxidase-conjugated secondary antibody (DakoCytomation, Carpinteria, CA, USA) for 1 h at room temperature. Immunoreactive polypeptide bands were visualized by using an enhanced chemiluminescence detection system, followed by exposure to X-ray film. Densitometry was performed by using a FluorChem documentation program (Alpha Innotech Corp., San Leandro, CA, USA) to measure the eNOS and GAPDH integrated density values (IDVs) of the aortic rings for each experimental condition.

### 2.5. Rat Aortic Endothelial Cell Culture

Primary rat aortic endothelial cells were cultured as previously described [25]. Briefly, aortas were isolated, cut open longitudinally, and digested with 0.2% collagenase directed against type 1A collagen (Sigma-Aldrich) for 15 min in a 37°C shaking water bath. RPMI medium supplemented with 10% fetal bovine serum and 1% penicillin/streptomycin was added to the cell suspension, and the cell suspension was then centrifuged at 1500 ×g for 10 min. The cell pellet was resuspended in the supplemented RPMI medium, and the cells were plated into culture flask (25 cm^2^) and allowed to settle for 1 h. At this time, the medium was changed to remove nonadherent cells. After the adherent cells reached 80% confluence, they were seeded onto coverslips in 6-well trays, pretreated with various drugs as described below, exposed to H_2_O_2_ (200 *μ*M, 60 min), and subjected to DHE-mediated fluorescence microscopy for the detection of H_2_O_2_-generated intracellular ROS/O_2_^∙−^.

### 2.6. ROS/O_2_^∙−^ Detection via DHE-Mediated Fluorescence Microscopy

Intracellular ROS/O_2_^∙−^ levels were examined in the primary rat aortic endothelial cells by using DHE-mediated fluorescence microscopy. The oxidation of DHE by intracellular ROS releases ethidium, which binds to DNA to emit fluorescence. The cells were pretreated at 37°C with TMP (10 nM, 60 min), apocynin (10 *μ*M, 30 min), DPI (10 nM, 30 min), Tiron (100 *μ*M) plus DETCA (1 mM) (30 min), or untreated control (60 min); exposed to H_2_O_2_ (200 *μ*M, 60 min); and then incubated with DHE (5 *μ*M, 20 min). Tiron, an O_2_^∙−^ scavenger, and DETCA, an O_2_^∙−^ generator, were utilized to confirm the production of O_2_^∙−^ in the endothelial cells following exposure to H_2_O_2_.

After incubation with DHE, the cells were rinsed with phosphate buffered saline, and the fluorescence of DNA-bound ethidium was measured under a FluoView Confocal Microscope (Olympus, Center Valley, PA, USA). The microscope was equipped with a 585-nm long pass filter and operated at an excitation wavelength of 515 nm. The fluorescence intensity of each sample was analyzed by using FluoView FV10-ASW software, version 1.5 (Olympus). The summarized data represent fold-changes in fluorescence intensity relative to that of the control, which was set to a value of 100%.

### 2.7. Statistical Analysis

All quantifiable data are expressed as the mean ± the standard error of the mean (SEM). GraphPad Prism software, version 6.0 (GraphPad Software, Inc., La Jolla, CA, USA), was used for data analysis. The vasorelaxing effect of TMP and other drugs was expressed as the percentage reduction of the Phe-evoked contraction. Nonlinear regression curve fitting was performed on individual cumulative Ach concentration-response curves to estimate the *E*_max⁡_ (maximal response) and the pD_2_ (negative logarithm of the EC_50_, where the EC_50_ represents the drug concentration that induces 50% of *E*_max⁡_) for each experimental agent (H_2_O_2_ or drug) or combination of agents. Thus, *E*_max⁡_ is a measure of effectiveness and pD_2_ is a measure of potency.

The concentration-response curves were analyzed by two-way analysis of variance (ANOVA) followed by the Bonferroni post hoc test. The statistical significance of the Western blotting data and the DHE-mediated fluorescence microscopy data was determined by one-way ANOVA followed by the Bonferroni post hoc test. In each case, a *P* value of less than 0.05 was considered significant.

## 3. Results

### 3.1. TMP Protects against H_2_O_2_-Provoked Endothelial Dysfunction

**Figure 1:**
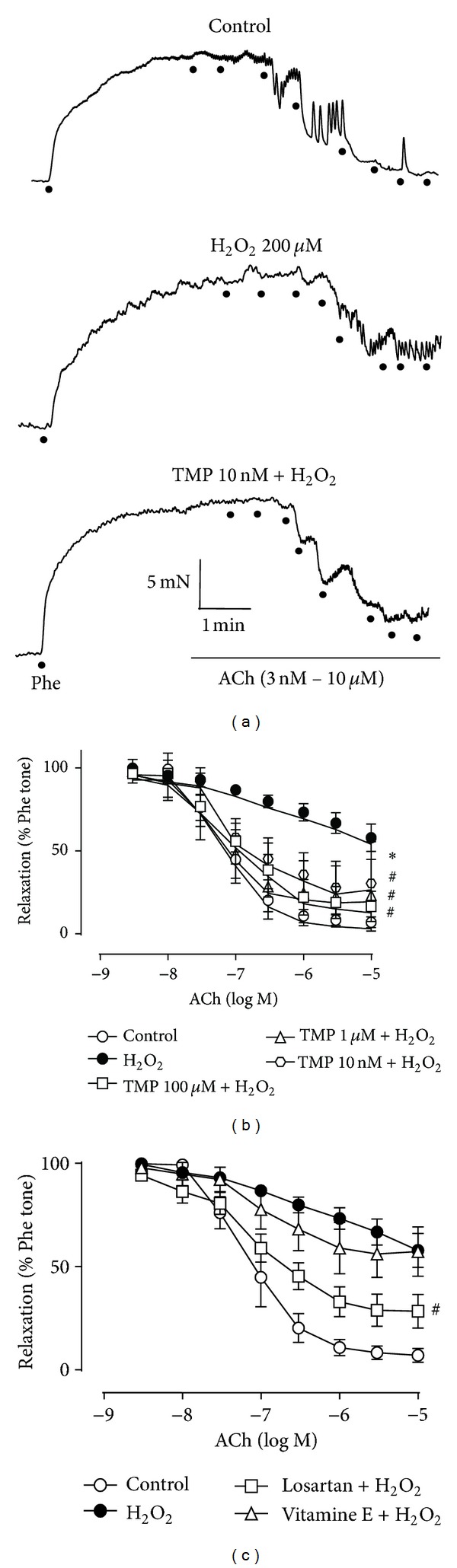
Tetramethylpyrazine (TMP) protects against hydrogen peroxide- (H_2_O_2_-) induced endothelial dysfunction. Phenylephrine (Phe, 0.3 *μ*M) was utilized to provoke contraction in isolated rat aortic rings, and acetylcholine (ACh, 3 nM–10 *μ*M) was utilized to induce EDR in the Phe-contracted rings. (a) Representative traces of ACh-induced EDR in H_2_O_2_ (200 *μ*M)-exposed rings, with and without TMP (10 nM) pretreatment, and control rings. (b), (c) Effects of various drug treatments on H_2_O_2_-provoked disruption of ACh-induced EDR. The graphs share the same data for the control and H_2_O_2_-treated groups. Data represent the mean ± the standard error of the mean (SEM) of 4–6 independent experiments (*P < 0.05 versus control; ^#^ *P* < 0.05 versus H_2_O_2_).

Phe (0.3 *μ*M) was utilized to provoke contraction in aortic rings isolated from adult Sprague Dawley rats, while ACh was employed to induce EDR in the Phe-contracted rings. ACh-induced EDR was markedly impaired in the rat aortic rings after 60 min exposure to H_2_O_2_ (200 *μ*M) (Table 1, Figure 1). However, TMP pretreatment for 60 min at concentrations of 10 nM, 1 *μ*M, and 100 *μ*M significantly reversed the endothelial impairment provoked by H_2_O_2_. TMP and losartan, the newly identified antioxidants, showed similar effects at 1 *μ*M (Table 1), while pretreatment with 100 *μ*M vitamin E, a classical antioxidant, did not greatly reverse the endothelial damage (Table 1, Figure 1).

### 3.2. H_2_O_2_-Provoked Endothelial Dysfunction Is Reversed by Catalase and NADPH Oxidase Inhibitors

**Figure 2:**
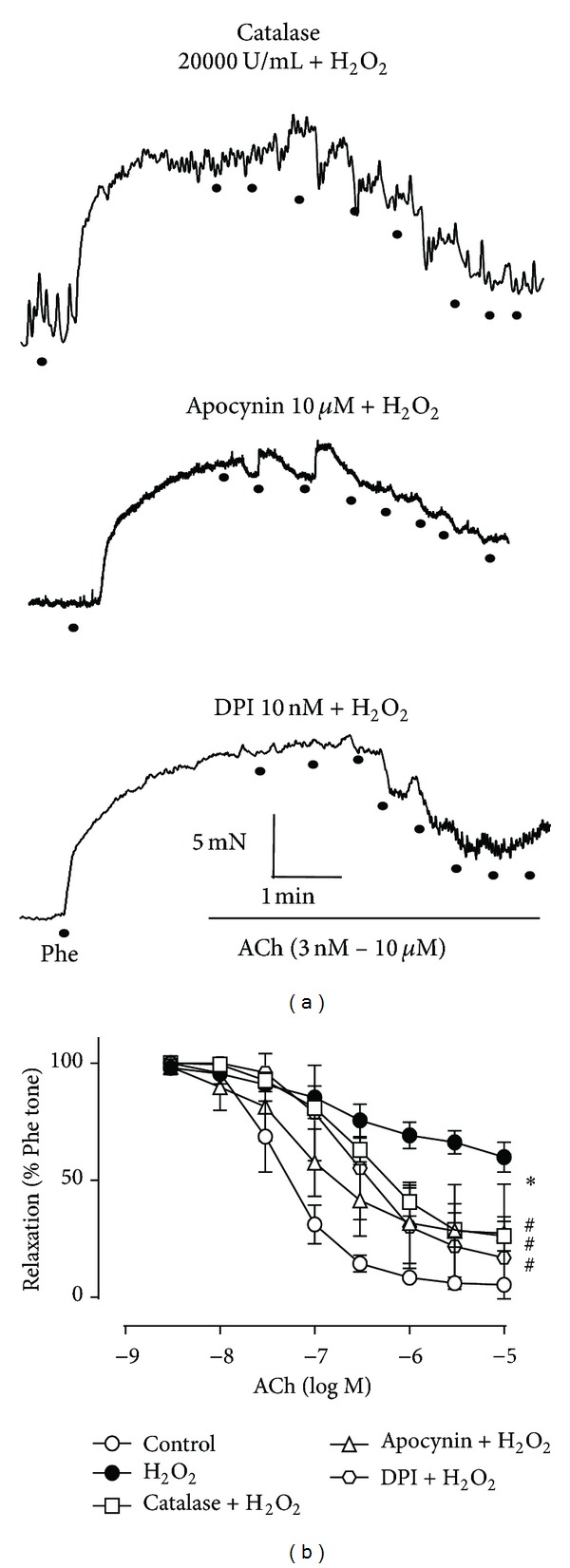
Hydrogen peroxide (H_2_O_2_)-induced endothelial dysfunction is reversed by catalase and NADPH oxidase inhibitors. (a) Representative traces of acetylcholine- (ACh-) induced endothelium-dependent relaxation (EDR) in H_2_O_2_- (200 *μ*M) treated rings with catalase (20000 U/mL), apocynin (10 *μ*M), or diphenyleneiodonium (DPI, 10 nM) pretreatment. (b) Effects of catalase, apocynin, and DPI on H_2_O_2_-provoked disruption of ACh-induced EDR. Data represent the mean ± the standard error of the mean (SEM) of 4–6 independent experiments (*P < 0.05 versus control; ^#^ *P* < 0.05 versus H_2_O_2_).

Catalase is a H_2_O_2_ scavenger that reduces H_2_O_2_ levels by converting the superoxide anions into water and molecular oxygen. Catalase (20000 U/mL, 30 min) pretreatment of rat aortic rings significantly prevented the H_2_O_2_-mediated disruption of ACh-induced EDR, albeit only partially (Table 2, Figure 2), suggesting that H_2_O_2_ exerts both direct and indirect effects to induce endothelial dysfunction. In support of this hypothesis, separate pretreatment of the rings with the NADPH oxidase inhibitors, apocynin (10 *μ*M, 30 min) and DPI (10 nM, 30 min), also significantly blocked the induction of vascular dysfunction by H_2_O_2_ (Table 2, Figure 2).

### 3.3. TMP Does Not Rescue EDR by Altering eNOS Expression

**Figure 3:**
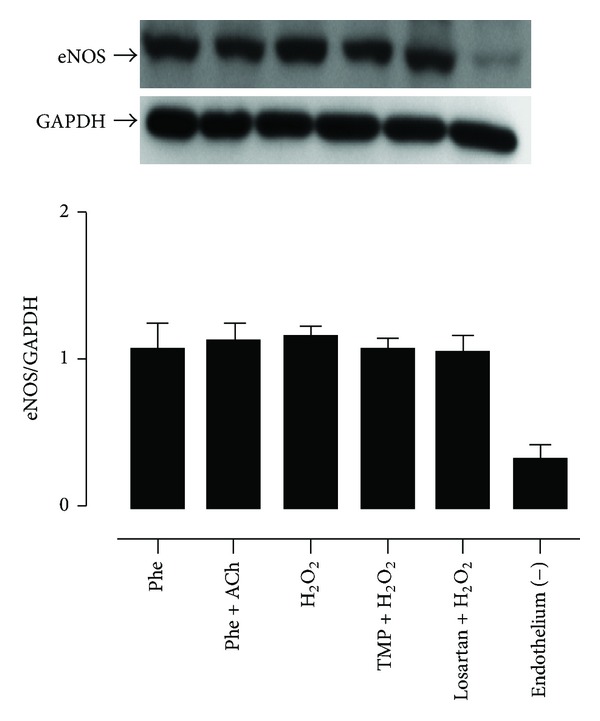
Tetramethylpyrazine (TMP) does not rescue endothelium-dependent relaxation (EDR) through actions on endothelial nitric oxide synthase (eNOS) expression. Rat aortic rings were subjected to various experimental conditions (phenylephrine (Phe); Phe plus acetylcholine (ACh); hydrogen peroxide (H_2_O_2_, 200 *μ*M); TMP (10 nM) plus H_2_O_2_; losartan (1 *μ*M) plus H_2_O_2_; or removal of the endothelium). The bar graph shows the ratio of the eNOS integrated density value (IDV) to the glyceraldehyde 3-phosphate dehydrogenase (GAPDH) IDV for each experimental condition. Data represent the mean ± the standard error of the mean (SEM) of 5–8 independent experiments.

Nitric oxide acts as a vasodilator and is produced through the actions of eNOS in the endothelium. Therefore, we reasoned that agents with the capacity to influence the EDR in rat aortic rings might have an effect on eNOS expression. However, none of the experimental conditions employed (Phe; Phe plus ACh; H_2_O_2_ (200 *μ*M, 60 min); TMP (10 nM, 60 min) plus H_2_O_2_; or losartan (1 *μ*M, 30 min) plus H_2_O_2_) had any effect on eNOS expression  in aortic rings with an intact endothelium, as assessed on Western blots (Figure 3) and by comparing the eNOS IDV/GAPDH IDV ratio for each experimental condition (Table 3, Figure 3). On the other hand, the endothelium-denuded group with no drug treatment showed significantly decreased eNOS expression relative to any group with an intact endothelium (Table 3, Figure 3).

### 3.4. TMP, NADPH Oxidase Inhibitors, and the ROS Scavenger, Tiron, Prevented the Production of O_2_^∙−^ in H_2_O_2_-Exposed Rat Aortic Endothelial Cells

**Figure 4:**
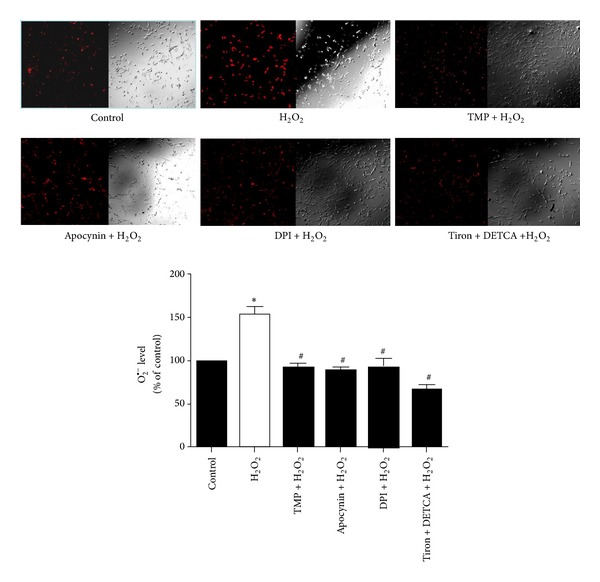
Tetramethylpyrazine (TMP), apocynin, diphenyleneiodonium (DPI), and Tiron prevent hydrogen peroxide- (H_2_O_2_-) induced production of the superoxide anion (O_2_^∙−^) in primary rat aortic endothelial cells. O_2_^∙−^ production was detected by the dihydroethidium- (DHE-) mediated fluorescence microscopy assay. Cells were treated with H_2_O_2_ (200 *μ*M) alone or in combination with TMP (10 nM), apocynin (10 *μ*M), DPI (10 nM), or Tiron (10 nM) plus diethyldithiocarbamic acid (DETCA) (1 mM) pretreatment. The bar graph shows the fluorescence intensity (%) of each experimental group relative to the control. Data represent the mean ± the standard error of the mean (SEM) of 3–9 independent experiments (*P < 0.05 versus control; ^#^ *P* < 0.05 versus H_2_O_2_).

Finally, we evaluated the ROS/O_2_^∙−^ content in primary cultures of H_2_O_2_-exposed rat aortic endothelial cells with and without drug pretreatment, because O_2_^∙−^ is a highly damaging species linked to substantial cytotoxicity in vascular endothelial cells. H_2_O_2_ (200 *μ*M, 60 min) triggered substantial ROS generation compared with the control group, as determined by a significant increase in fluorescence intensity attributable to ethidium-bound DNA. However, the fluorescence intensity was significantly blocked by pretreatment with TMP (10 nM, 60 min), apocynin (10 *μ*M, 30 min), DPI (10 nM, 30 min), or Tiron (an ROS scavenger) (100 *μ*M, 30 min) plus DETCA (a superoxide dismutase inhibitor that increases intracellular O_2_^∙−^ content) (1 mM, 30 min) (Table 4, Figure 4).

## 4. Discussion

Vascular events (death from all vascular causes, nonfatal stroke, or nonfatal myocardial infarction) are complex occurrences caused by multiple factors, and ROS-induced endothelial injury is suggested to be the common denominator in most vascular conditions [26]. Consequently, antioxidant therapy, which directly targets the common mediator of vascular disorders, shows great promise for the prevention and management of cardio-cerebrovascular diseases.

The current study explored the capacity of acute pretreatment with TMP, an antioxidant constituent of Rhizoma Ligustici Wallichii, to overturn the H_2_O_2_-mediated impairment of ACh-induced relaxation in the rat aorta. We found that the exposure of rat aortic rings to TMP prior to H_2_O_2_ treatment did indeed thwart the actions of H_2_O_2_ to promote endothelial dysfunction (Table 1, Figure 1). TMP was effective at surprisingly low doses (i.e., nM levels), yet vitamin E, a classical antioxidant, did not provide the same protective benefits at a considerably higher concentration (100 *μ*M) (Table 1, Figure 1). This may be due to the use of high concentration of H_2_O_2_ (200 *μ*M) to induce impaired EDR and the potent antioxidant effect of TMP. The protective effect of TMP by reduction of oxidative stress in the present study is consistent with other studies working in different systems [12, 13, 27–29].

H_2_O_2_ is a relatively stable ROS with dual functions in the vasculature, depending on its effective concentration. For example, low levels of H_2_O_2_ maintain the physiology of vascular endothelial cells [30], and H_2_O_2_ seldom exerts vascular damage in the absence of unpaired electrons [31]. Therefore, drugs which are capable of stimulating superoxide dismutase to transform O_2_^∙−^ into oxygen and H_2_O_2_ are regarded as protective agents [32]. However, increasing evidence demonstrates that substantial exposure to H_2_O_2_ at high concentrations (i.e., >100 *μ*M) contributes to vascular injury and associated inflammatory responses, vascular endothelial cell cytotoxicity and apoptosis, and even vascular events [33]. The present study therefore exposed the isolated rat aortic rings to high levels of H_2_O_2_ (200 *μ*M) for 60 min, with the goal of establishing an* in vitro* model of oxidative stress-induced endothelial dysfunction. Given that ACh-induced relaxation is endothelium-dependent and reflects normal endothelium functionality [34], H_2_O_2_-induced disruption of ACh-stimulated EDR mimics the pathophysiology of ROS-triggered vascular diseases.

Interestingly, TMP or losartan pretreatment of H_2_O_2_-exposed aortic rings did not rescue EDR by altering eNOS expression (Table 3, Figure 3), although H_2_O_2_ reportedly affects endothelial function by modulating Enos content in the rabbit aorta [35]. In particular, low levels of the ROS exert vasorelaxing effects via eNOS upregulation of nitric oxide [35], suggesting that high concentrations of H_2_O_2_, such as that used herein, might impair EDR by downregulating eNOS content. Nonetheless, this was not the case (Table 3, Figure 3). The protective effect of TMP to H_2_O_2_-induced endothelial dysfunction in the present study may be due to the reported TMP-mediated activation of eNOS activity through PI3K/Akt and phosphorylation of eNOS at Ser1177 for increased generation of NO in myocardial ischaemia reperfusion [12]. At the same time, TMP can alleviate oxidative stress by increased total antioxidant activity and SOD1 activity [13]. Furthermore, catalase, a strong H_2_O_2_ scavenger [21], only partially reversed the impairment of EDR by H_2_O_2_ (Table 2, Figure 2). Hence, H_2_O_2_ may indirectly as well as directly harm the vascular endothelium.

An earlier investigation indicated that H_2_O_2_ can activate NADPH oxidase to produce O_2_^∙−^ *in vitro* [36]. We therefore pretreated rat aortic rings with two different NADPH inhibitors, apocynin and DPI, prior to H_2_O_2_ exposure and found that the drugs significantly safeguarded the aortas from ROS insult, and rescued EDR (Table 2, Figure 2). We also conducted DHE-mediated fluorescence measurements in cultured primary rat aortic endothelial cells to investigate the impact of H_2_O_2_ and various drugs on intracellular O_2_^∙−^ content. As a result, H_2_O_2_ enhanced O_2_^∙−^ levels, while pretreatment with TMP, apocynin, DPI, or Tiron plus DETCA prevented this action (Table 4, Figure 4). This exciting finding implies that O_2_^∙−^ is produced in response to exposure of the vascular endothelium to oxidative stress, which is potentially mediated via the stimulation of NADPH oxidase. On the other hand, it is still possible that H_2_O_2_ exposure to rat aortic rings may increase ROS generation from mitochondria due to increased oxidative stress. TMP could improve the impaired EDR by reduction of mitochondrial ROS production [27]. We cannot exclude this possible mechanism although its contributory role may not be significant. At the same time, it has been shown that the antioxidant effect of TMP likely is due to its ability of the mitochondrial biogenesis [28].

The possibility that TMP averts H_2_O_2_-provoked endothelial dysfunction by inhibiting NADPH oxidase is supported by our findings that its actions were similar to those of apocynin and DPI in both isolated aortic rings (compare Table 1 with Figure 1 and Table 2 with Figure 2) and primary aortic endothelial cells (Table 4, Figure 4). NADPH oxidase is a key generator of ROS in blood vessel walls during the progression of vascular disease [37]. An unequivocal demonstration of the TMP-mediated suppression of NADPH oxidase expression/activity under pathological conditions, with a concomitant reduction in the overproduction of O_2_^∙−^ and other ROS, would undeniably strengthen our proposal that TMP might serve as a new antioxidant agent for the management of vascular diseases. However, this hypothesis requires further investigation, especially in regard with determining the NADPH oxidase and O_2_^∙−^ levels in rat aortic rings post-TMP treatment.

Other possible alternative mechanisms may be offered by TMP to protect endothelial dysfunction. The protective effect of TMP may be due to its action to induce anti-inflammatory effect in endothelium. TMP has been shown to reverse the decrease of NO production induced by TNF-α and inhibit the downregulated expression of intracellular adhesion molecular-1 and heat shock protein 60 mediated by TNF-α, suggesting that TMP may protect endothelium through inhibition of immunological reactions [29]. In addition, TMP treatment has been shown to prevent the increment of both inducible NO synthase and TNF-α expression [38], improving survival of rodent model of endotoxic shock induced by lipopolysaccharide [39]. TMP has also been demonstrated to protect streptozotocin-induced diabetic rats by downregulated expression of vascular endothelial growth factor [40].

## 5. Conclusions

In conclusion, the present study showed that TMP prevented oxidative stress-induced endothelial dysfunction in isolated rat aortic rings without affecting eNOS expression. In addition, TMP reduced O_2_^∙−^ accumulation in H_2_O_2_-exposed primary rat aortic endothelial cells. The NADPH oxidase inhibitors, apocynin and DPI, displayed the same actions as TMP in both* in vitro* models. These results suggest that TMP might find therapeutic utility as an efficacious antioxidant for the treatment of human cardio-cerebrovascular diseases and warrant further exploration of its actions in whole-animal models and preclinical studies. However, the role of TMP in inhibiting NADPH oxidase remains to be elucidated.

## Figures and Tables

**Table 1:** Effects of TMP, losartan, and vitamin E on H_2_O_2_-provoked endothelial dysfunction in isolated rat aortic rings.

Treatment	pD_2_	*E*_max⁡_ (%)	*N*
Control	7.20 ± 0.12	6.97 ± 3.32	4
H_2_O_2_ (200 *μ*M)	6.40 ± 0.23∗	57.86 ± 8.24∗	6
TMP (100 *μ*M) + H_2_O_2_	7.05 ± 0.22^#^	16.59 ± 9.15^#^	5
TMP (1 *μ*M) + H_2_O_2_	7.29 ± 0.17^#^	23.31 ± 9.69^#^	5
TMP (10 nM) + H_2_O_2_	7.07 ± 0.15^#^	30.38 ± 11.25	5
Losartan (1 *μ*M) + H_2_O_2_	7.02 ± 0.17^#^	28.36 ± 8.15^#^	5
Vitamin E (100 *μ*M) + H_2_O_2_	6.94 ± 0.37	57.23 ± 11.97	5

Data represent the mean ± the SEM of 4–6 independent experiments (*P < 0.05 versus control; ^#^ *P* < 0.05 versus H_2_O_2_).

*E*_max⁡_: maximal response; H_2_O_2_: hydrogen peroxide; pD_2_: negative logarithm of the EC_50_; SEM: standard error of the mean; TMP: tetramethylpyrazine.

**Table 2:** Effects of catalase, apocynin, and DPI on H_2_O_2_-provoked endothelial dysfunction in isolated rat aortic rings.

Treatment	pD_2_	*E*_max⁡_ (%)	*N*
Control	7.22 ± 0.07	5.32 ± 2.93	4
H_2_O_2_ (200 *μ*M)	4.63 ± 0.25∗	59.88 ± 6.44∗	6
Catalase (20000 U/mL) + H_2_O_2_	6.10 ± 0.07^#^	26.11 ± 6.34^#^	4
Apocynin (10 *μ*M) + H_2_O_2_	6.51 ± 0.13^#^	27.31 ± 10.51^#^	4
DPI (10 nM) + H_2_O_2_	6.33 ± 0.09^#^	16.88 ± 8.82^#^	4

Data represent the mean ± the SEM of 4–6 independent experiments (*P < 0.05 versus control; ^#^ *P* < 0.05 versus H_2_O_2_).

DPI: diphenyleneiodonium; *E*_max⁡_: maximal response; H_2_O_2_: hydrogen peroxide; pD_2_: negative logarithm of the EC_50_; SEM: standard error of the mean.

**Table 3:** eNOSIDV/GAPDH IDV ratios in isolated rat aortic rings.

Treatment	Ratio	*N*
Phe (0.3 *μ*M)	1.10 ± 0.08	5
Phe + ACh (10 *μ*M)	1.24 ± 0.04	8
H_2_O_2_ (200 *μ*M)	1.13 ± 0.03	8
TMP (10 nM ) + H_2_O_2_	1.01 ± 0.03	6
Losartan (1 *μ*M ) + H_2_O_2_	1.13 ± 0.05	6
Endothelium removal	0.32 ± 0.04	6

Data represent the mean ± the SEM of 5–8 independent experiments.

ACh: acetylcholine; eNOS: endothelial nitric oxide synthase; GADPH: glyceraldehyde 3-phosphate dehydrogenase; H_2_O_2_: hydrogen peroxide; IDV: integrated density value; Phe: phenylephrine; TMP: tetramethylpyrazine.

**Table 4:** DHE-mediated fluorescence microscopy assay in primary rat aortic endothelial cells.

Treatment	Fluorescence intensity (% of control)	*N*
Control	100 ± 0	7
H_2_O_2_ (200 *μ*M)	158 ± 1.16∗	9
TMP (10 nM) + H_2_O_2_	86 ± 2.10^#^	5
Apocynin (10 *μ*M) + H_2_O_2_	88.30 ± 2.25^#^	4
DPI (10 nM) + H_2_O_2_	87.28 ± 5.40^#^	3
Tiron (100 *μ*M) + DETCA (1 mM) + H_2_O_2_	67.95 ± 2.81^#^	4

Data represent the means ± the SEM of 3–9 independent experiments (*P < 0.05 versus control; ^#^ *P* < 0.05 versus H_2_O_2_).

DHE: dihydroethidium; DETCA: diethyldithiocarbamic acid; DPI: diphenyleneiodonium; H_2_O_2_: hydrogen peroxide; SEM: standard error of the mean; TMP: tetramethylpyrazine.

## Abbreviations

Ach: Acetylcholine
ANOVA: Analysis of variance
DETCA: Diethyldithiocarbamic acid
DHE: Dihydroethidium
DPI: Diphenyleneiodonium
EDR: Endothelium-dependent relaxation
*E*_max⁡_: Maximal response
eNOS: Endothelial nitric oxide synthase
GAPDH: Glyceraldehyde 3-phosphate dehydrogenase
H_2_O_2_: Hydrogen peroxide
IDV: Integrated density value
O_2_^∙−^: Superoxide anion
pD_2_: Negative logarithm of the EC_50_ (drug concentration that induces 50% of the *E*_max⁡_)
Phe: Phenylephrine
ROS: Reactive oxygen species
SDS: Sodium dodecyl sulfate
SEM: Standard error of the mean
SOD: Superoxide dismutase
TMP: Tetramethylpyrazine.
